# Moving toward Narrowing the United States Gap in Assisted Reproductive Technology (ART) Racial and Ethnic Disparities in the Next Decade

**DOI:** 10.3390/jcm13082224

**Published:** 2024-04-11

**Authors:** Jasmin Mahabamunuge, David B. Seifer

**Affiliations:** Department of Obstetrics, Gynecology, and Reproductive Sciences, Yale University School of Medicine, 333 Cedar Street, New Haven, CT 06510, USA; david.seifer@yale.edu

**Keywords:** race, ethnicity, disparities, DART hypothesis, bias, assisted reproductive technology, in vitro fertilization, intrauterine insemination, access, treatment outcomes, public health

## Abstract

The Disparities in Assisted Reproductive Technology (DART) hypothesis, initially described in 2013 and further modified in 2022, is a conceptual framework to examine the scope and depth of underlying contributing factors to the differences in access and treatment outcomes for racial and ethnic minorities undergoing ART in the United States. In 2009, the World Health Organization defined infertility as a disease of the reproductive system, thus recognizing it as a medical problem warranting treatment. Now, infertility care is largely recognized as a human right. However, disparities in Reproductive Endocrinology and Infertility (REI) care in the US persist today. While several studies and review articles have suggested possible solutions to racial and ethnic disparities in access and outcomes in ART, few have accounted for and addressed the multiple complex factors contributing to these disparities on a systemic level. This review aims to acknowledge and address the myriad of contributing factors through the DART hypothesis which converge in racial/ethnic disparities in ART and considers possible solutions to effect large scale societal change by narrowing these gaps within the next decade.

## 1. Introduction

In 2009, the World Health Organization defined infertility as a disease of the reproductive system, thus recognizing it as a medical problem warranting treatment [1]. Since then, infertility care has become recognized as a human right in the US and internationally [1,2,3]. However, disparities in Reproductive Endocrinology and Infertility (REI) care in the US persist [4]. In recent decades, inequities in infertility care have become a topic of actionable interest. In 2020, the American Society for Reproductive Medicine (ASRM) expressed concern regarding racial and ethnic disparities affecting patients seeking or in need of fertility care and, accordingly, created a dedicated diversity, equity and inclusion (DEI) taskforce [5]. While healthcare disparities in the US exist based on numerous sociodemographic factors, including but not limited to income, availability of insurance, geography, sexual and gender identity, they are notably significant when broken down by race/ethnicity, rippling into multiple clinical contexts [6].

Racial/ethnic disparities in infertility care are multifactorial; they can often begin long before patients present for infertility care or qualify for treatment and funnel into Assisted Reproductive Technology (ART) outcomes [7]. This is evidenced by decreased contraceptive knowledge among Black and Hispanic veteran patients, particularly regarding an awareness of the irreversible nature of tubal sterilization, decreased rates of HPV vaccination, decreased antimullerian hormone levels/ovarian reserves at a given age, increased tubal factor disease and fibroid burden, and increased rates of sterilization at the time of cesarean section in racial/ethnic minorities [8,9,10,11,12,13]. Additionally, the rate of infertility is higher in minority populations, with Black women having at least 1.5 times the rate of infertility as White women, in addition to increased rates of comorbidities and risk factors associated with infertility [14]. In this review, we will focus specifically on ART outcomes, with the understanding that disparities are often at play prior to needing or meeting the criteria for specialized infertility care. As it currently stands, non-White race is an independent poor prognostic factor in infertility care. However, race is believed to be a social construct, and does not necessarily reflect biological differences between groups [15,16]. As such, this review offers a comprehensive series of suggestions by reviewing and discussing previous publications through the framework of the Disparities in ART (DART) hypothesis. Through this hypothesis, we focus on factors which contribute to racial/ethnic disparities, and discuss ways to potentially narrow the gap in access and treatment outcomes in ART within the next 10 years.

## 2. Current Disparities in ART

Prior to exploring new and previously proposed interventions intended to narrow the disparity gap in ART, it is prudent to discuss the pervasiveness of racial/ethnic disparities, specifically in ART access and treatment outcomes. Also, it is necessary to build this discussion by reiterating that rates of infertility are higher in minority populations, with Black women having the highest rates of infertility and, therefore, the highest need for infertility care [14].

Though use of in vitro fertilization (IVF) has tripled in the last 20 years, this trend is disparate for racial/ethnic minority groups [6,17]. In an observational study evaluating utilization of infertility services in the US by race/ethnicity, using data from the National Survey of Family Growth cycles from 2002, 2006–2010 and 2011–2013, based on participant responses regarding use of infertility services, the disparities in utilization were underscored. Despite higher rates of infertility, Black, Hispanic, and American Indian/Alaska Native patients are less likely to utilize medical assistance to achieve pregnancy compared to White patients. After adjusting for relevant covariates, this difference persists in Black patients only, with Black women exhibiting a 23% lower prevalence of medical assistance in becoming pregnant [18]. Differential utilization of ART can be quite stark, with some studies showing up to 70% lower prevalence in Black women in regional versus national studies, respectively [6,19]. In a retrospective cohort study examining the relationship between race/ethnicity and the utilization of different infertility treatments using the United States (US) birth data files from 2011 to 2019, non-Hispanic Black and Hispanic women had about a 70% lower likelihood of receiving any infertility treatment, compared to non-Hispanic White women. Furthermore, non-Hispanic White women were the most represented group for live births associated with any type of infertility treatment (53.2%) and non-Hispanic Black women were the least represented (3.7%) [6].

Oocyte cryopreservation, an REI outcome that is typically independent from an infertility diagnosis, is less common in racial and ethnic minorities. In a retrospective cohort analysis using the Society for Assisted Reproductive Technology Clinical Outcome Reporting System for patients undergoing oocyte cryopreservation from 2012 to 2016, oocyte cryopreservation was least common in Black and Hispanic patients [20,21]. Of the cycles with reported race/ethnicity data, 66.5% were performed in White patients, 9.6% in Asian patients, 7.1% in Black patients, and 4.5% in Hispanic patients. Interestingly, oocyte yield was comparable across ethnic groups, with the mean (standard deviation) of oocytes retrieved per cycle equaling 12.9 (9.7) for White patients, 13.2 (11.4) for Black patients, 10.6 (8.4) for Asian patients, 12.1 (9.9) for Hispanic patients, and 13.7 (10.3) for patients who identified as another race [21]. This re-demonstrates that disparities in REI care are present even prior to the diagnosis of infertility and the need for infertility treatment amongst racial/ethnic minorities.

For many patients, additional barriers emerge even after presenting to care which, in turn, limits the ability to provide quality care. In a retrospective study of 87 patients seeking fertility care at a single resident/fellow REI clinic in New York from 2020 to 2022, 88.5% of participants identified as non-White and most had Medicaid; 70–80% completed their lab evaluation; 59.8% were able to complete a scheduled HSG; and only 27.6% of patients’ partners completed a semen analysis [22]. While more research is needed to fully elucidate the likely multi-factorial etiology of increased incomplete workups in racial/ethnic minority patients, transportation, cultural/social stigma, and financial constraints have been identified as contributing factors [23].

Accessing and completing care are certainly not the only barriers contributing to racial/ethnic inequities in ART utilization. Implicit bias, seen with differential fertility counseling in young Black and Latina women compared to White women newly diagnosed with cancer, and insurance reimbursement models or the lack thereof, are examples of pervasive systemic and structural bias contributing to this disparity [7,24,25]. While studies show referrals for fertility preservation are notably low in general, they vary by race/ethnicity, highlighting the role provider bias plays in perpetuating racial/ethnic disparities in ART. In a retrospective cohort study of women aged 18–42 years diagnosed with a new breast, gynecologic, hematologic or gastrointestinal cancer at a single institution between 2008 and 2010, the odds of a fertility preservation consultation referral were about two times higher for White women, compared to Black, Hispanic and Asian women [26]. Patients are aware and concerned about differential or inferior treatment, as studies show Black and Hispanic women face more difficulty finding a fertility physician with whom they feel comfortable, thus leading to delays in workup and treatment [27,28]. Provider bias and its detrimental effects on patient health continue to be evaluated and publicized [29]. In 2020, The American College of Obstetrics and Gynecology published a collective action addressing racism as a joint statement recognizing historical, societal, institutional and practitioner factors contributing to inequities in obstetrics and gynecology [30].

Racial/ethnic disparities also persist in ART treatment outcomes [31]. For example, despite higher access and utilization of fertility care by Black women in a military population, lower ART success and decreased live birth rates were seen; which was, in part, attributed to an increased fibroid burden in Black women [32]. Additionally, in a study of women undergoing autologous in vitro fertilization (IVF) from 2010 to 2012, Black and Asian women had lower odds of clinical intrauterine pregnancy and live birth, and higher rates of spontaneous abortion compared with White women [33]. Similarly, studies consistently show a decrease in live birth rates for Black women undergoing autologous, non-donor, fresh embryo transfers compared to White women, after controlling for multiple factors. Live birth rates are additionally lower for Black patients undergoing frozen embryo transfers [34,35,36,37]. Racial differences are also seen with intrauterine insemination (IUI). In a retrospective analysis of patients undergoing IUI from 2007 to 2012, American Indian patients had 66% lower pregnancy rates compared to non-Hispanic Whites when patient and cycle characteristics were controlled for [38]. Differences in live birth rates are likely multi-factorial and, in part, attributable to other comorbid diseases that occur at a higher rate in minority populations, such as tubal factor disease, uterine factor disease, and elevated BMI. In a retrospective cohort study with 1110 patients undergoing 2254 autologous IVF cycles between 2014 and 2019 at an academic fertility center in the Southeastern United States, the neighborhood deprivation index, a proxy for socioeconomic and environmental factors was not statistically significantly associated with the live birth rate. Live birth per cycle was lower among Black (24%) compared to White patients (32%), and the crude probability of miscarriage per clinical pregnancy was higher among Black patients (22%) compared to White patients (12%) [39].

Biological disparities are seen at the level of ovarian function with decreased age-related ovarian reserves observed in Black women compared to White, Asian and Hispanic women [40,41]. Additionally, despite greater ovarian responsiveness, Black and Hispanic patients have lower live birth rates compared with White patients, though this was not statistically significantly different after adjusting for confounders [42]. Furthermore, birth rates remain lower for Black women, even when using vitrified oocytes from healthy donors [43]. Racial/ethnic differences are also seen in hormone production, metabolism, and endometrial receptivity, ultimately contributing to worse outcomes in minority populations. For example, in a retrospective cohort study of 3289 ART cycles conducted between 2009 and 2013 at the Shady Grove Reproductive Science Center, premature progesterone elevation on the last day of ART stimulation was shown to have a negative effect on live birth rates. Additionally, the prevalence of elevated progesterone on the last day of ART stimulation was higher in Black, Asian and Hispanic women, compared to White women [44]. Lastly, even when live births are achieved, perinatal outcomes are persistently worse for racial/ethnic minority groups with higher rates of gestational diabetes, fetal growth restriction, preterm labor, preeclampsia and type II diabetes postpartum [45,46,47].

## 3. What Has Been Proposed?

Some of the previously suggested solutions/approaches to mitigating inequities in ART have focused on cost burden and legislation. In models where ART is more affordable, such as in the military, Black women demonstrated a fourfold increase in utilization of ART [48]. Currently, 21 states provide some amount of insurance infertility coverage in the US, which is frequently limited to infertility workup and evaluation. Treatment coverage is often limited in terms of amount and type of treatment, however. For instance, IVF is often excluded from these mandates, and when IVF is included, a trial of IUI is typically required before only a limited number of IVF cycles become eligible [49]. State insurance mandates have been suggested to make ART more affordable and thus more accessible, as comprehensive mandates have been associated with reduced disparities in ART utilization in Hispanic and non-Hispanic Black populations [35]. However, more recent evidence shows that while states with mandated coverage for fertility diagnosis and treatment have seen an increase in access to ART in all racial groups, especially for Asian patients, outcomes remain unchanged [50,51]. So far, the current state mandates for donor oocyte ART have been insufficient for decreasing racial/ethnic disparities [52,53]. This is in part due to exemptions, which in turn present additional obstacles for otherwise eligible patients. Certainly, this has been the case in Massachusetts, which has provided mandated coverage for IVF since 1987 [54]. Despite this, exemptions exist for those enrolled with self-insured, employer-sponsored health plans, Medicare and/or Medicaid, OPM-affiliated health plans, and TRICARE, making this benefit inaccessible to many patients in Massachusetts [54]. In fact, only 26.2–36.0% of Massachusetts-based reproductive-age women comprised eligible beneficiaries of the Massachusetts Infertility Insurance Mandate during the 2016–2019 study period.

Moreover, recent publications have suggested addressing the mismatch in supply and demand of the infertility provider pipeline in ART by recommending expansion of much-needed clinical services to other non-REI trained providers [55,56]. Additionally, telehealth utilization and resident/fellow run fertility clinics have been suggested in previous studies and reviews as solutions to increase accessibility of infertility care and bridge disparities [57,58]. To narrow the existing national racial/ethnic disparities in access and treatment outcomes in ART within the next decade, we suggest possible solutions by approaching the challenges of disparity in care through the prism of the DART hypothesis.

## 4. Pathways for Accelerated Change—DART Hypothesis Revisited

The DART hypothesis in racial and ethnic disparities in access and outcomes of IVF treatment in the US was initially proposed by Seifer et al., 2013, in a book chapter entitled “Toward a Better Understanding of Racial Disparities in Utilization and Outcomes of IVF Treatment in the USA,” and further revisited and revised in 2022 [59,60]. This approach calls for identifying, integrating, and addressing the multiple factors contributing to racial/ethnic disparities in ART.

The prohibiting factors at play prior to patients presenting for fertility care provide an opportune area for potential improvement. Educating patients about reproductive health, fertility, and the prevalence of age-related infertility, as well as proper utilization of ART, may help to mitigate stigma and shame, which likely contribute to delayed presentation in patients from racial/ethnic minority backgrounds [27,61]. This delay may exacerbate the already well-known present challenge of age-related infertility [62,63]. Timely referral by OB-GYN generalists and primary care to a fertility specialist is highly encouraged for women 35 or older after 6 months of unprotected intercourse and immediate referral for women 40 or older to not exacerbate the impact of age-related infertility. Beyond normalizing the timetable for referral for specialized fertility care, increased education may yield improved utilization due to a better understanding of reproductive health, disease prevention, and an increased awareness of potential insurance options available. Seeking an explanation of insurance benefits early on may assist those who have insurance to understand their options of pursuing appropriate treatment in a more timely manner. Possible time-sensitive intervention points may include high school, when sexual and reproductive health are often introduced into educational curricula, college, and at various timepoints in community centers, as studies show there is a need for increased education among reproductive aged women. In a cross-sectional study including 1127 participants, a validated fertility awareness survey entitled the Fertility & Infertility Treatment Knowledge Score was administered to 18–45 year old reproductive aged women in the US, and revealed a mean score of 55.9%, indicating an overall low fertility awareness [64]. During educational interventions, emphasis should simultaneously be placed on the reproductive lifespan, diseases contributing to subfertility and infertility, treatment/prevention, and when to seek care with a fertility specialist concurrently with general reproductive health education. Similarly, co-morbidities contributing to subfertility and infertility in racial/ethnic minority populations may necessitate aggressive treatment to address the higher disease burden contributing to poorer ART outcomes.

Moreover, recruiting fertility providers from diverse racial, ethnic, and cultural backgrounds is essential [65]. Healthcare studies show patients generally fare better when care was provided by more diverse teams. In a 2019 umbrella review of systematic reviews and meta analyses, positive associations were noted between diversity, quality, and financial performance in the healthcare environment [66]. In addition to mitigating societal and educational disparities, this may assuage patient concerns regarding comfort with and understanding from providers, a known factor delaying presentation to care for racial/ethnic minorities, which likely exacerbates the negative impact of age-related infertility [27,59,67]. Additional benefits of increasing fertility provider diversity are a theoretical increase in availability and accessibly of providers, particularly in underserved areas, as travel and travel distance contribute to increased rates of discontinuation of care after unsuccessful IVF cycles in minority patients, even in the case of insurance coverage [68]. Lastly, recruitment of diverse fertility providers will help address the shortage of REI trained physicians in the US. In a 2009 review of the economic impact of assisted reproductive technology, it was estimated that North America meets only 24% of its ART needs [69]. Increasing diversity within the REI workforce, therefore, will simultaneously address the national shortage of REI providers and the lack of diversity among REI providers. Leveraging other non-REI trained providers, including general obstetrician/gynecologists and advanced practice practitioners, has also been proposed to address the widening supply and demand mismatch for REI providers and delivers yet another possible solution for patients from racial/ethnic minority backgrounds to access timely care [55,56]. Educational tools and guidelines, such as practice bulletins (AGOC Committee Opinion No. 781) and hospital pathways, for non-REI trained providers may further bolster this possible option [70,71]. More research is indicated to assess the effectiveness of the above interventions in reducing racial/ethnic disparities in ART.

Additional actionable items emerge when patients overcome the obstacles of presenting to care. First, this includes using tailored evidence-based ART approaches for patients from racial/ethnic minority backgrounds, particularly when prior “standard of care” attempts have failed. Additional research in this area of racial/ethnic disparities is encouraged to further consider and customize care for patients who fail “standard of care” therapies. ART methods with more equitable outcomes should also be prioritized, when appropriate. For instance, in a retrospective cohort study including patients with infertility undergoing IVF with Intracytoplasmic Sperm Injection (ICSI), preimplantation genetic testing for aneuploidy (PGT-A), and an autologous single euploid embryo transfer (SEET) from 2015 to 2019 at a single private and academic assisted center, there was no difference in euploid or live birth rates based on self-reported race [72]. However, as is discussed below, these additional tests and methodologies can be costly and thus prohibitive. Therefore, such testing is of highest utility in applicable populations, and shared decision making with patients is recommended. Third, mandatory implicit bias training for providers is encouraged to improve the patient–provider relationship, particularly as it pertains to cultural competence, understanding and addressing patient mistrust, and combating implicit bias [5,30,73].

For many racial/ethnic minorities, the cost of IVF is prohibitive. IVF in the US is costly, with standard IVF cycles starting at $12,500 USD. Many patients are not prepared or able to spend 50% of their disposable income on IVF, which is often required to cover this cost [69]. In a systematic review using data from 40 studies in high-income countries from 2011 to 2022, ART interventions were examined using an economic evaluation component. Specifically, the study identified the most common high-cost interventions not necessarily adding to care. This included preimplantation genetic testing for aneuploidy (PGT-A) for the general population and ICSI for unexplained infertility [74]. Therefore, access to fertility care can additionally be expanded at the level of the provider or practice for racial/ethnic minority patients by avoiding unnecessary testing, where applicable.

Addressing systems-based, institutional and societal contributors to racial/ethnic disparities in ART is required for meaningful and durable change [75]. In addition to the above suggestions, funding, and legislation changes are needed. Policy reform may be warranted to implement and expand state and federal mandates for insurance coverage of fertility care. Though insurance mandates alone have demonstrated to be insufficient in bridging the gap in racial/ethnic disparities in ART, more comprehensive insurance mandates, as opposed to limited or no mandates, confer more equitable access [50,53,76,77]. Second, institutions providing fertility care should be encouraged to review outcomes stratified by sociodemographic factors, including race/ethnicity, to truly identify and address disparities on an institutional level. This will also facilitate introspection and self-remediation at an institutional level. Third, increased allocation of resources from the public and private sector are of utmost importance to continue identifying, understanding, and ultimately narrowing the racial/ethnic disparities gap in ART. Lastly, currently only 66% of ART cycles have race/ethnicity information completed. Policies to strongly encourage and incentivize recording of patient self-reported race/ethnicity data could be implemented resulting in greater effort for annual reporting of ART cycles to SARTCORS and thus increase opportunities for disparities research [35,78].

## 5. Conclusions

In summary, a multi-pronged approach is encouraged in the next decade to narrow and ultimately close the racial access and treatment disparity gap in reproductive medicine. Future efforts focused on enhancing provider cultural competency, patient and community education regarding timely referral for evaluation and the challenge of coping with age-related infertility, advocacy for broadening greater insurance coverage, and more favorable public healthcare policies are likely to narrow the racial/ethnic disparity gap in ART access and treatment outcomes in the next 10 years.

## 6. Future Directions

**Figure 1:**
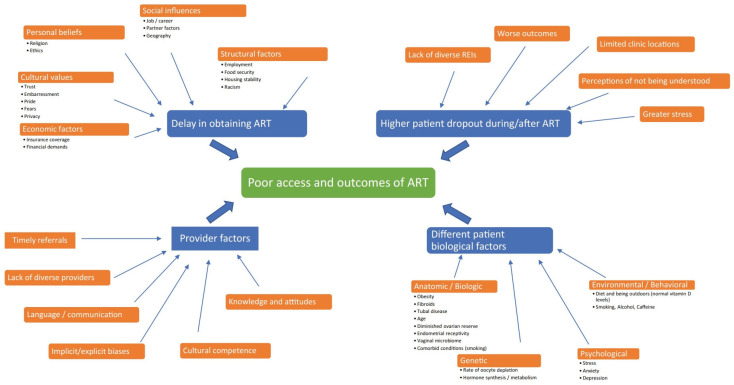
The DART hypothesis in racial and ethnic disparities in access and outcomes of IVF treatment in the US. Published with permission from Reproductive Sciences [60].

Racial and ethnic disparities in reproductive medicine are a result of multiple complex factors. Novel integrated and multifaceted solutions are needed to comprehensively address racial/ethnic disparities in ART. This review of the DART hypothesis provides one such framework of the myriad contributing factors converging in current racial/ethnic disparities in ART, and provides suggested solutions to achieve large-scale change and a narrowing of this gap in the next decade (Figure 1 and Table 1).

## Figures and Tables

**Table 1:** List of suggested solutions to narrow the racial/ethnic disparity gap in ART access and treatment outcomes in the next decade.

Problem	Solution
Financial obstacles leading to decreased care utilization	Expand comprehensive insurance mandates to cover fertility care/treatment.
Delayed presentation due to insufficient understanding of age-related infertility and available resources	Immediate referral to REI specialist for women 35 or older who have had 6 months of unprotected intercourse and immediate referral for women 40 or older. Increase funding from private and public sectors for: early education at the high school, college and/or community level. Emphasize prevention and treatment of co-morbidities contributing to infertility.
Delayed presentation for care due to stigma and shame	Destigmatize subfertility and infertility with early education and discussion of available resources.
Delayed presentation due to difficulty finding providers patients are comfortable with	Increase recruitment of diverse providers. Mandatory provider bias training.
Provider bias and inconsistent counseling	Mandatory provider bias training.
Inconsistent follow up due to burden of travel outside of underserved areas	Recruit diverse providers to increase number of providers in underserved areas. Increase counseling and workup done by non-REI providers to improve access and address delays in care. Provide non-REI providers education and resources to best care for patients.
Inadequate research identifying and addressing factors contributing to poorer outcomes	Incentivize SART clinics to record self-report race/ethnic in ART national databases. Increase funding for disparities research. Increase recruitment of racial/ethnic minorities in clinical studies.
Poorer ART outcomes in racial/ethnic minorities	All the above, plus: Prioritize evidence-based ART methods that work for racial/ethnic minorities when the “standard of care” fails. Encourage institutions providing fertility care to stratify outcomes by race/ethnicity to facilitate introspection and self-remediation. Continue to identify and address systemic and institutional contributors.

## References

[1] Zegers-Hochschild F., Adamson G.D., De Mouzon J., Ishihara O., Mansour R., Nygren K., Sullivan E., Van Der Poel S. (2009). The International Committee for Monitoring Assisted Reproductive Technology (ICMART) and the World Health Organization (WHO) Revised Glossary on ART Terminology, 2009. Hum. Reprod..

[2] Shah P.K., Gher J.M. (2023). Human Rights Approaches to Reducing Infertility. Int. J. Gynecol. Obstet..

[3] United Nations. General Assembly (1948). Universal Declaration of Human Rights.

[4] Ethics Committee of the American Society for Reproductive Medicine (2021). Disparities in Access to Effective Treatment for Infertility in the United States: An Ethics Committee Opinion. Fertil. Steril..

[5] ASRM Task Force on Diversity, Equity and Inclusion. Statement of Interest and Concern. https://www.asrm.org/globalassets/_asrm/about-us/committees/asrm-dei-task-force-report-11-30-2020.pdf.

[6] Dongarwar D., Mercado-Evans V., Adu-Gyamfi S., Laracuente M.-L., Salihu H.M. (2022). Racial/Ethnic Disparities in Infertility Treatment Utilization in the US, 2011–2019. Syst. Biol. Reprod. Med..

[7] Beroukhim G., Mahabamunuge J., Pal L. (2022). Racial Disparities in Access to Reproductive Health and Fertility Care in the United States. Curr. Opin. Obstet. Gynecol..

[8] Rosenfeld E., Callegari L.S., Sileanu F.E., Zhao X., Schwarz E.B., Mor M.K., Borrero S. (2017). Racial and Ethnic Disparities in Contraceptive Knowledge among Women Veterans in the ECUUN Study. Contraception.

[9] Buck DiSilvestro J., Ulmer K.K., Hedges M., Kardonsky K., Bruegl A.S. (2024). Cervical Cancer. Obstet. Gynecol. Clin. N. Am..

[10] Jin S.W., Lattimore D.C., Harlin E., Davis L., Erholtz V., Brandt H.M. (2023). Medical and Public Health Professionals’ Perceived Facilitators and Barriers of Human Papillomavirus (HPV) Vaccination among African American Adolescents in Shelby County, Tennessee. BMC Health Serv. Res..

[11] Garcia G., Richardson D.M., Gonzales K.L., Cuevas A.G. (2015). Trends and Disparities in Postpartum Sterilization after Cesarean Section, 2000 through 2008. Women’s Health Issues.

[12] Bleil M.E., Gregorich S.E., Adler N.E., Sternfeld B., Rosen M.P., Cedars M.I. (2014). Race/Ethnic Disparities in Reproductive Age: An Examination of Ovarian Reserve Estimates across Four Race/Ethnic Groups of Healthy, Regularly Cycling Women. Fertil. Steril..

[13] Seifer D.B., Zackula R., Grainger D.A. (2010). Trends of Racial Disparities in Assisted Reproductive Technology Outcomes in Black Women Compared with White Women: Society for Assisted Reproductive Technology 1999 and 2000 vs. 2004–2006. Fertil. Steril..

[14] Craig L.B., Peck J.D., Janitz A.E. (2019). The Prevalence of Infertility in American Indian/Alaska Natives and Other Racial/Ethnic Groups: National Survey of Family Growth. Paediatr. Perinat. Epidemiol..

[15] Brown T.H., Homan P. (2024). Structural Racism and Health Stratification: Connecting Theory to Measurement. J. Health Soc. Behav..

[16] Smedley A., Smedley B.D. (2005). Race as Biology Is Fiction, Racism as a Social Problem Is Real: Anthropological and Historical Perspectives on the Social Construction of Race. Am. Psychol..

[17] Center for Disease Control and Prevention (2023). 2021 National ART Summary.

[18] Janitz A.E., Peck J.D., Craig L.B. (2019). Racial/Ethnic Differences in the Utilization of Infertility Services: A Focus on American Indian/Alaska Natives. Matern. Child Health J..

[19] Chin H.B., Howards P.P., Kramer M.R., Mertens A.C., Spencer J.B. (2015). Racial Disparities in Seeking Care for Help Getting Pregnant. Paediatr. Perinat. Epidemiol..

[20] Gadson A.K., Sauerbrun-Cutler M.-T., Eaton J.L. (2024). Racial Disparities in Fertility Care: A Narrative Review of Challenges in the Utilization of Fertility Preservation and ART in Minority Populations. J. Clin. Med..

[21] Katler Q.S., Shandley L.M., Hipp H.S., Kawwass J.F. (2021). National Egg-Freezing Trends: Cycle and Patient Characteristics with a Focus on Race/Ethnicity. Fertil. Steril..

[22] Tarrash M., Kuyoro O., Goldman R., Mullin C. (2024). Characteristics of Patients Seeking Fertility Care in a Low-Income Setting. JBRA Assist. Reprod..

[23] Mackay A., Taylor S., Glass B. (2023). Inequity of Access: Scoping the Barriers to Assisted Reproductive Technologies. Pharmacy.

[24] Letourneau J.M., Smith J.F., Ebbel E.E., Craig A., Katz P.P., Cedars M.I., Rosen M.P. (2012). Racial, Socioeconomic, and Demographic Disparities in Access to Fertility Preservation in Young Women Diagnosed with Cancer: Fertility Preservation Disparities. Cancer.

[25] Turner K.A., Spurlin E.E., Jimenez P.T. (2023). Disparities in Female Oncofertility Care in the United States: More Questions Than Answers. Life.

[26] Goodman L.R., Balthazar U., Kim J., Mersereau J.E. (2012). Trends of Socioeconomic Disparities in Referral Patterns for Fertility Preservation Consultation. Hum. Reprod..

[27] Missmer S.A., Seifer D.B., Jain T. (2011). Cultural Factors Contributing to Health Care Disparities among Patients with Infertility in Midwestern United States. Fertil. Steril..

[28] Blair I.V., Steiner J.F., Fairclough D.L., Hanratty R., Price D.W., Hirsh H.K., Wright L.A., Bronsert M., Karimkhani E., Magid D.J. (2013). Clinicians’ Implicit Ethnic/Racial Bias and Perceptions of Care Among Black and Latino Patients. Ann. Fam. Med..

[29] Gonzalez C.M., Ark T.K., Fisher M.R., Marantz P.R., Burgess D.J., Milan F., Samuel M.T., Lypson M.L., Rodriguez C.J., Kalet A.L. (2024). Racial Implicit Bias and Communication Among Physicians in a Simulated Environment. JAMA Netw. Open.

[30] American College of Obstetrics and Gynecology (2020). Joint Statement: Collective Action Addressing Racism.

[31] Beroukhim G., Seifer D.B. (2023). Racial and Ethnic Disparities in Access to and Outcomes of Infertility Treatment and Assisted Reproductive Technology in the United States. Endocrinol. Metab. Clin. N. Am..

[32] Feinberg E.C., Larsen F.W., Catherino W.H., Zhang J., Armstrong A.Y. (2006). Comparison of Assisted Reproductive Technology Utilization and Outcomes between Caucasian and African American Patients in an Equal-Access-to-Care Setting. Fertil. Steril..

[33] McQueen D.B., Schufreider A., Lee S.M., Feinberg E.C., Uhler M.L. (2015). Racial Disparities in In Vitro Fertilization Outcomes. Fertil. Steril..

[34] Csokmay J.M., Hill M.J., Maguire M., Payson M.D., Fujimoto V.Y., Armstrong A.Y. (2011). Are There Ethnic Differences in Pregnancy Rates in African-American versus White Women Undergoing Frozen Blastocyst Transfers?. Fertil. Steril..

[35] Seifer D.B., Simsek B., Wantman E., Kotlyar A.M. (2020). Status of Racial Disparities between Black and White Women Undergoing Assisted Reproductive Technology in the US. Reprod. Biol. Endocrinol..

[36] Heyward Q., Walter J.R., Alur-Gupta S., Lal A., Berger D.S., Koelper N., Butts S.F., Gracia C.R. (2021). Racial Disparities in Frozen Embryo Transfer Success. J. Assist. Reprod. Genet..

[37] Makhijani R., Godiwala P., Grady J., Christy A., Thornton K., Grow D., Engmann L. (2022). Black Race Associated with Lower Live Birth Rate in Frozen-Thawed Blastocyst Transfer Cycles: An Analysis of 7,002 Society for Assisted Reproductive Technology Frozen-Thawed Blastocyst Transfer Cycles. Fertil. Steril..

[38] Craig L.B., Weedin E.A., Walker W.D., Janitz A.E., Hansen K.R., Peck J.D. (2018). Racial and Ethnic Differences in Pregnancy Rates Following Intrauterine Insemination with a Focus on American Indians. J. Racial Ethn. Health Disparities.

[39] Andre K.E., Hood R.B., Gaskins A.J., Kawwass J.F., Almquist R.G., Kramer M.R., Hipp H.S. (2024). Neighborhood Deprivation and Racial Differences in In Vitro Fertilization Outcomes. Am. J. Obstet. Gynecol..

[40] Dimitriadis I., Batsis M., Petrozza J.C., Souter I. (2017). Racial Disparities in Fertility Care: An Analysis of 4537 Intrauterine Insemination Cycles. J. Racial Ethn. Health Disparities.

[41] Seifer D.B., Golub E.T., Lambert-Messerlian G., Benning L., Anastos K., Watts D.H., Cohen M.H., Karim R., Young M.A., Minkoff H. (2009). Variations in Serum Müllerian Inhibiting Substance between White, Black, and Hispanic Women. Fertil. Steril..

[42] Lee I.T., Berger D.S., Koelper N., Senapati S., Mainigi M. (2023). Race, Ovarian Responsiveness, and Live Birth after In Vitro Fertilization. Fertil. Steril..

[43] Liu Y., Hipp H.S., Nagy Z.P., Capelouto S.M., Shapiro D.B., Spencer J.B., Gaskins A.J. (2021). The Effect of Donor and Recipient Race on Outcomes of Assisted Reproduction. Am. J. Obstet. Gynecol..

[44] Hill M.J., Royster G.D., Taneja M., Healy M.W., Zarek S.M., Christy A.Y., DeCherney A.H., Widra E., Devine K. (2017). Does Elevated Progesterone on Day of Oocyte Maturation Play a Role in the Racial Disparities in IVF Outcomes?. Reprod. Biomed. Online.

[45] McKenzie-Sampson S., Baer R.J., Jelliffe-Pawlowski L.L., Karasek D., Riddell C.A., Torres J.M., Blebu B.E. (2024). Structural Racism, Nativity and Risk of Adverse Perinatal Outcomes among Black Women. Paediatr. Perinat. Epidemiol..

[46] Janevic T., McCarthy K., Liu S.H., Huyhn M., Kennedy J., Tai Chan H., Mayer V.L., Vieira L., Tabaei B., Howell F. (2023). Racial and Ethnic Inequities in Development of Type 2 Diabetes After Gestational Diabetes Mellitus. Obstet. Gynecol..

[47] Garland C.E., Geller S.E., Koch A.R. (2024). Adverse Delivery and Neonatal Outcomes Among Women with Severe Maternal Morbidity in Illinois, 2018–2019. J. Women’s Health.

[48] McCarthy-Keith D.M., Schisterman E.F., Robinson R.D., O’Leary K., Lucidi R.S., Armstrong A.Y. (2010). Will Decreasing Assisted Reproduction Technology Costs Improve Utilization and Outcomes among Minority Women?. Fertil. Steril..

[49] Kawwass J.F., Penzias A.S., Adashi E.Y. (2021). Fertility—A Human Right Worthy of Mandated Insurance Coverage: The Evolution, Limitations, and Future of Access to Care. Fertil. Steril..

[50] Korkidakis A., DeSantis C.E., Kissin D.M., Hacker M.R., Koniares K., Yartel A., Adashi E.Y., Penzias A.S. (2024). State Insurance Mandates and Racial and Ethnic Inequities in Assisted Reproductive Technology Utilization. Fertil. Steril..

[51] Vu M., Stuehling D., Li D., Alur-Gupta S. (2023). Fertility Care for All: Impact of New York State’s Medicaid Expansion on Infertility Care. J. Assist. Reprod. Genet..

[52] Liao C., Kotlyar A.M., Seifer D.B. (2023). Effect of State Insurance Mandates on Racial/Ethnic Disparities in the Utilization and Outcomes of Donor Oocyte–Assisted Reproductive Technologies. Fertil. Steril..

[53] Correia K.F.B., Kraschel K., Seifer D.B. (2023). State Insurance Mandates for In Vitro Fertilization Are Not Associated with Improving Racial and Ethnic Disparities in Utilization and Treatment Outcomes. Am. J. Obstet. Gynecol..

[54] Koniares K.G., Penzias A.S., Roosevelt J., Adashi E.Y. (2022). The Massachusetts Infertility Insurance Mandate: Not Nearly Enough. FS Rep..

[55] Hariton E., Alvero R., Hill M.J., Mersereau J.E., Perman S., Sable D., Wang F., Adamson G.D., Coutifaris C., Craig L.B. (2023). Meeting the Demand for Fertility Services: The Present and Future of Reproductive Endocrinology and Infertility in the United States. Fertil. Steril..

[56] Adeleye A.J., Kawwass J.F., Brauer A., Storment J., Patrizio P., Feinberg E. (2023). The Mismatch in Supply and Demand: Reproductive Endocrinology and Infertility Workforce Challenges and Controversies. Fertil. Steril..

[57] Brodeur T.Y., Grow D., Esfandiari N. (2022). Access to Fertility Care in Geographically Underserved Populations, a Second Look. Reprod. Sci..

[58] Blakemore J.K., Maxwell S.M., Hodes-Wertz B., Goldman K.N. (2020). Access to Infertility Care in a Low-Resource Setting: Bridging the Gap through Resident and Fellow Education in a New York City Public Hospital. J. Assist. Reprod. Genet..

[59] Seifer D.B., Sharara F.I., Jain T., Sharara F.I. (2013). Toward a Better Understanding of Racial Disparities in Utilization and Outcomes of IVF Treatment in the USA. Ethnic Differences in Fertility and Assisted Reproduction.

[60] Seifer D.B., Sharara F.I., Jain T. (2022). The Disparities in ART (DART) Hypothesis of Racial and Ethnic Disparities in Access and Outcomes of IVF Treatment in the USA. Reprod. Sci..

[61] Fujimoto V.Y., Jain T., Alvero R., Nelson L.M., Catherino W.H., Olatinwo M., Marsh E.E., Broomfield D., Taylor H., Armstrong A.Y. (2010). Proceedings from the Conference on Reproductive Problems in Women of Color. Fertil. Steril..

[62] Delbaere I., Verbiest S., Tydén T. (2020). Knowledge about the Impact of Age on Fertility: A Brief Review. Ups. J. Med. Sci..

[63] Chua S.J., Danhof N.A., Mochtar M.H., Van Wely M., McLernon D.J., Custers I., Lee E., Dreyer K., Cahill D.J., Gillett W.R. (2020). Age-Related Natural Fertility Outcomes in Women over 35 Years: A Systematic Review and Individual Participant Data Meta-Analysis. Hum. Reprod..

[64] Kudesia R., Chernyak E., McAvey B. (2017). Low Fertility Awareness in United States Reproductive-Aged Women and Medical Trainees: Creation and Validation of the Fertility & Infertility Treatment Knowledge Score (FIT-KS). Fertil. Steril..

[65] Rayburn W.F. (2024). Diversity in Academic Obstetrics and Gynecology. Obstet. Gynecol. Clin. N. Am..

[66] Gomez L.E., Bernet P. (2019). Diversity Improves Performance and Outcomes. J. Natl. Med. Assoc..

[67] Galic I., Swanson A., Warren C., Negris O., Bozen A., Brown D., Lawson A., Jain T. (2021). Infertility in the Midwest: Perceptions and Attitudes of Current Treatment. Am. J. Obstet. Gynecol..

[68] Almquist R.G., Barrera C.M., Fried R., Boulet S.L., Kawwass J.F., Hipp H.S. (2022). Impact of Access to Care and Race/Ethnicity on IVF Care Discontinuation. Reprod. Biomed. Online.

[69] Chambers G.M., Sullivan E.A., Ishihara O., Chapman M.G., Adamson G.D. (2009). The Economic Impact of Assisted Reproductive Technology: A Review of Selected Developed Countries. Fertil. Steril..

[70] (2019). Infertility Workup for the Women’s Health Specialist: ACOG Committee Opinion, Number 781. Obstet. Gynecol..

[71] Polyzos N.P., Ayoubi J.M., Pirtea P. (2022). General Infertility Workup in Times of High Assisted Reproductive Technology Efficacy. Fertil. Steril..

[72] Alkon-Meadows T., Hernandez-Nieto C., Jackson-Bey T., Cacchione T.A., Lee J., Luna-Rojas M., Gounko D., Copperman A., Buyuk E. (2024). Correlation of Self-Reported Racial Background to Euploidy Status and Live Birth Rates in Assisted Reproductive Technology Cycles. J. Assist. Reprod. Genet..

[73] Gopal D.P., Chetty U., O’Donnell P., Gajria C., Blackadder-Weinstein J. (2021). Implicit Bias in Healthcare: Clinical Practice, Research and Decision Making. Future Healthc. J..

[74] Olive E., Bull C., Gordon A., Davies-Tuck M., Wang R., Callander E. (2024). Economic Evaluations of Assisted Reproductive Technologies in High-Income Countries: A Systematic Review. Hum. Reprod..

[75] Butts S.F. (2021). Health Disparities of African Americans in Reproductive Medicine. Fertil. Steril..

[76] Volovsky M., Seifer D.B. (2024). State Insurance Mandates Are Necessary but Not Sufficient for Closing the Racial and Ethnic Disparity Gap in Assisted Reproductive Technology. Fertil. Steril..

[77] Seifer D.B., Wantman E., Sparks A.E., Luke B., Doody K.J., Toner J.P., Van Voorhis B.J., Lin P.C., Reindollar R.H. (2018). National Survey of the Society for Assisted Reproductive Technology Membership Regarding Insurance Coverage for Assisted Reproductive Technologies. Fertil. Steril..

[78] Seifer D.B., Richard-Davis G., Alvero R. (2024). Closing the Gap on Racial Disparities—Increasing Race/Ethnicity Demographics Reporting in the SART CORS Registry. Fertil. Steril..

